# GExplore 1.5: a comprehensive *Caenorhabditis elegans* database for the analysis of gene function with a new user-friendly web interface

**DOI:** 10.1093/database/baaf044

**Published:** 2025-09-24

**Authors:** Harald Hutter, Mehrdad Moosavi, Nelly Mafi

**Affiliations:** Department of Biological Sciences, Simon Fraser University, 8888 University Drive Burnaby, BC V5A 1S6, Canada; Department of Biological Sciences, Simon Fraser University, 8888 University Drive Burnaby, BC V5A 1S6, Canada; Emily Carr University of Art and Design, 520 1st Ave E. Vancouver, BC V5T 0H2, Canada

## Abstract

GExplore is an online tool to assist with large-scale data mining of selected datasets related to gene and protein function in *Caenorhabditis elegans*. Here, we describe the current version GExplore 1.5, which contains new datasets and display options as well as a completely redesigned web interface. GExplore now consists of six databases. The gene database contains protein domain information, general expression, and phenotype data as well as interacting genes, gene ontology annotations, and disease associations. The mutation database contains a curated list of more than 200 000 mutations affecting the protein sequences of all protein-coding genes. The protein database contains proteome data from 19 different nematode species, four genetic model organisms and the human proteome for comparison. Three genome-scale RNAseq expression databases contain expression profiles of different developmental stages from embryo to adult, tissues-specific expression profiles at the L2 stage, and expression profiles of the major tissues in the developing embryo at five different time points from gastrulation to the beginning of terminal differentiation. The web-based user interface has been completely redeveloped for the current version. The search interfaces allow users to explore content of the individual databases in detail. The interactive display pages enable the user to fine-tune the results, display additional data, and download the results. GExplore is a tool to quickly obtain an overview of biological and biochemical functions of large groups of genes or identify genes with a certain combination of features for further experimental analysis.

**Database URL**: https://genome.science.sfu.ca/gexplore

## Introduction

### Background

Sequencing of the *Caenorhabditis elegans* genome in 1998 [1] revealed a surprisingly large number of genes (∼20 000). For most genes, functional data were not available at the time and there was an increasing demand for genome-scale data mining. The genome sequence provided information about protein sequences. The presence of certain protein domains, such as a kinase domain, allowed a functional grouping of genes and provided an overview of the sizes of various gene families. A major goal during the development of GExplore was to provide a search and display interface that allows a multi-gene display of selected datasets related to gene and protein function. The initial version of GExplore [2] contained data about the domain organization of all proteins, phenotype data from genome-wide RNAi experiments, and early genome-wide expression data. Gene and protein data in the GExplore databases are downloaded from WormBase [3], the central repository for data related to genetics and genomics of the nematode *C. elegans*. GExplore is designed to provide a multi-gene user interface to complement the single-gene user interface of WormBase, which is optimized for the display of all information related to one gene on a single webpage.

### Previous updates

Over time, additional datasets were included into the main ‘gene’ database of GExplore and additional databases were established [4]. The ‘mutation’ database was developed in response to large-scale genome sequencing efforts that led to an explosion of ‘variants’ in WormBase including many single nucleotide polymorphisms from natural isolates. Most of these are not expected to have any functional consequences. The GExplore mutation database contains a curated list of alleles affecting the amino acid sequence of proteins with potentially deleterious affects for the function of the protein. Most of these alleles alter exons causing missense or nonsense mutations or a loss of amino acids in the case of deletion alleles. Other mutations, such as those at splice junctions or deletions beginning or ending in introns, can lead to intronic sequences being translated into amino acid sequences and premature stop codons. The search page allows the user to select for certain types of mutations, e.g. nonsense alleles or deletions to identify putative null alleles or missense alleles affecting certain parts of the protein. The results page shows the location of each mutation mapped onto the domain organization of the protein. This allows a quick identification of available alleles in larger sets of genes.

When genome sequences became available for more species including many that have not been studied in the lab, we established a new protein database that initially contained the proteomes of nine nematodes species to allow comparisons of the proteomes and the identification of gene families based on protein domain features.

Currently, GExplore contains six databases that collectively provide a quick and user-friendly access to selected datasets to probe large sets of candidate genes for putative functions and/or prioritize genes for further analysis.

## Methods

### Gene data

Information about location, description, phenotype, expression, and gene ontology (GO) annotation for all protein-coding genes was downloaded using SimpleMine (http://www.wormbase.org/tools/simplemine). Data were assembled into database Tables using custom Perl scripts, which are available for download on the GExplore help page.

### Mutation data

The gff annotation file ‘c_elegans.PRJNA13758.WS296.annotations.gff3’ was downloaded from the WormBase FTP server (ftp://ftp.wormbase.org/pub/wormbase/). Information on the molecular nature and localization of *C. elegans* mutations was collected from this file using custom Perl scripts and incorporated into a MySQL database. Data processing scripts are available for download on the GExplore help page. WormMine was used to collect the names of all variants affecting protein coding genes. Variants extracted from the gff file were filtered to keep only mutations affecting the protein sequence by searching for one of the following keywords in the description: coding_sequence_variant, deletion, frameshift_variant, inframe_deletion, inframe_insertion, missense_variant, protein_altering_variant, splice_acceptor_variant, splice_donor_variant, splice_region_variant, start_lost, stop_gained, stop_lost, transcript_ablation.

### Proteome data

Protein sequences (file c_elegans.PRJNA13758.WS296.protein.fa) were downloaded from the WormBase FTP server (ftp://ftp.wormbase.org/pub/wormbase/) using release WS296. Domain predictions were done using SMART with the help of a batch processing script provided on the SMART [5] website (http://smart.embl-heidelberg.de/help/SMART_batch.pl). Custom Perl scripts were used to further process the raw SMART data files for incorporation into a MySQL database. Data processing scripts are available for download on the GExplore help page.

### Expression datasets

The expression data for the different stages of the life cycle of *C. elegans* are from [6]. The expression data for cells/tissues at the L2 stage are from [7] and the expression data for the embryonic tissues are from [8]. The raw data and images providing a graphic display of expression profiles were provided by the corresponding author, Dr Waterston.

## Results

**Figure 1.**
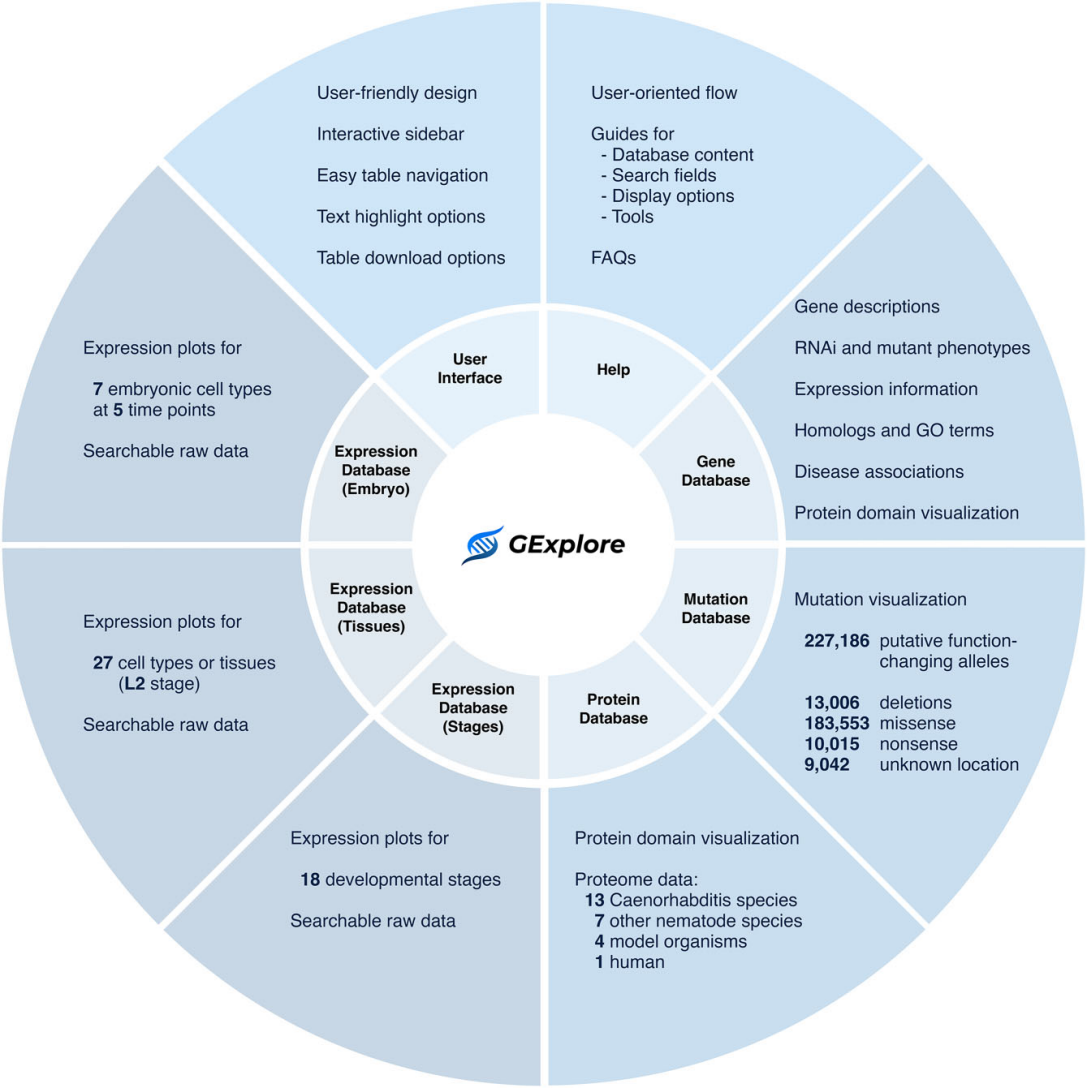
Overview of GExplore 1.5 showing the six databases—gene, mutation, protein, expression (stages), expression (tissues), and expression (embryo).

Since the last published update [4], we continued to expand the datasets available in GExplore. The current version includes updated datasets, additional databases, and several new features to improve data presentation, all within a fully redesigned, user-friendly website that improves accessibility (Fig. 1).

### Dataset updates, new databases, and new display features

#### Gene database

**Figure 2.**
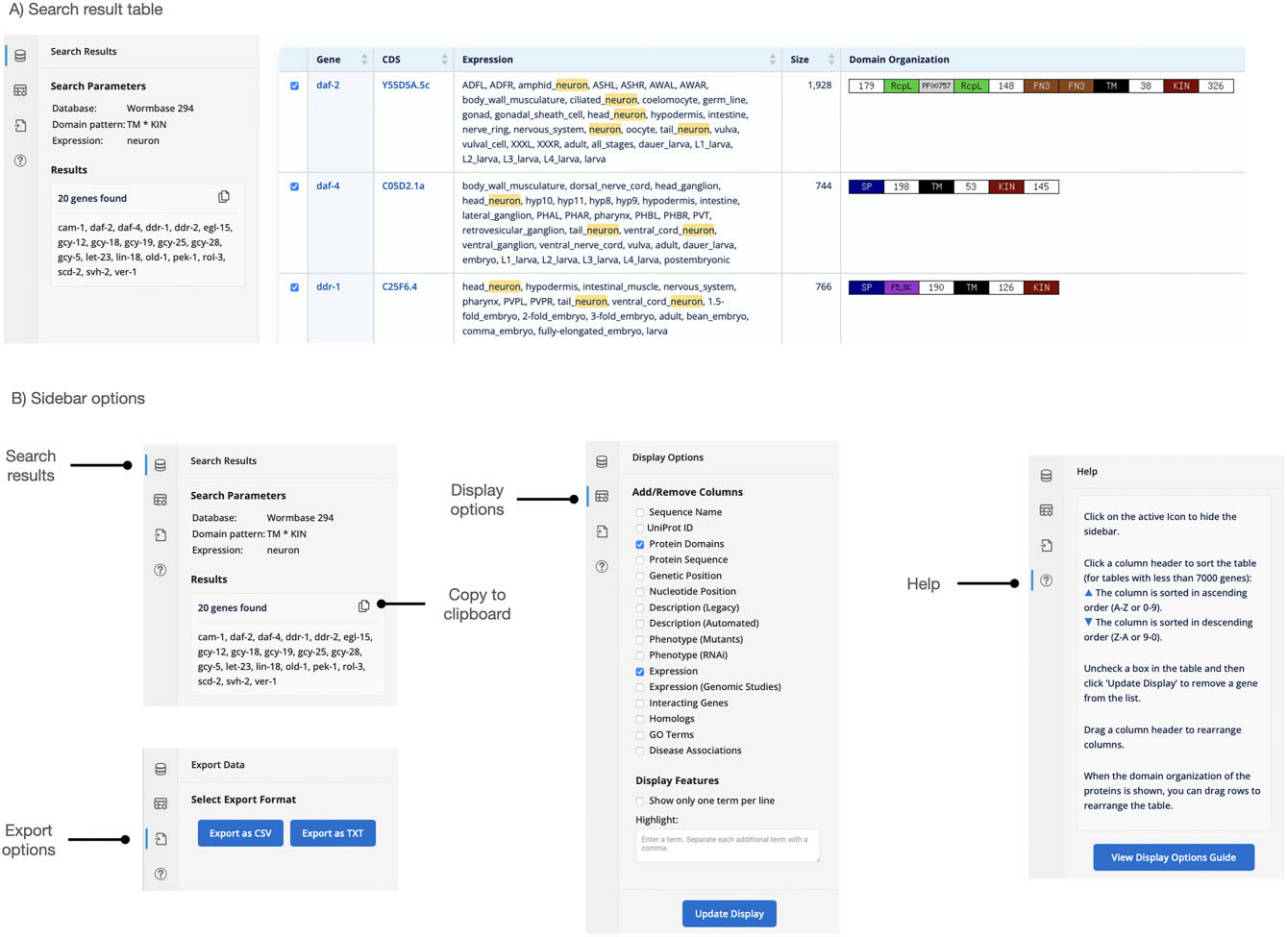
Display page of the gene database showing the results of a search for kinase receptors (proteins with a transmembrane domain and an intracellular kinase domain) that are expressed in neurons.

The main gene database was updated to the current WormBase release (WS296, April 2025). It now includes: searchable ‘legacy’ and ‘automated’ descriptions, RNAi and mutant phenotypes, general expression data and expression data from genome-scale studies, three classes of interacting genes (physical, genetic, and regulatory), GO term annotations and disease associations as well as the genetic and nucleotide positions of the genes. On the display page (Fig. 2A) users can show some or all the datasets mentioned above (independent of the initial search criteria). In addition, the homologs from the main genetic model organisms (*Saccharomyces cerevisiae, Drosophila melanogaster, Danio rerio*, and *Mus musculus*) as well as human homologs can be displayed. At the protein level, either the amino acid sequence or the domain organization visualization can be displayed. Search terms are highlighted in the result table for easier recognition and the user can interactively choose to highlight additional terms in the result table. This provides an interactive interface that can help to refine downstream searches.

#### Mutation database

**Figure 3.**
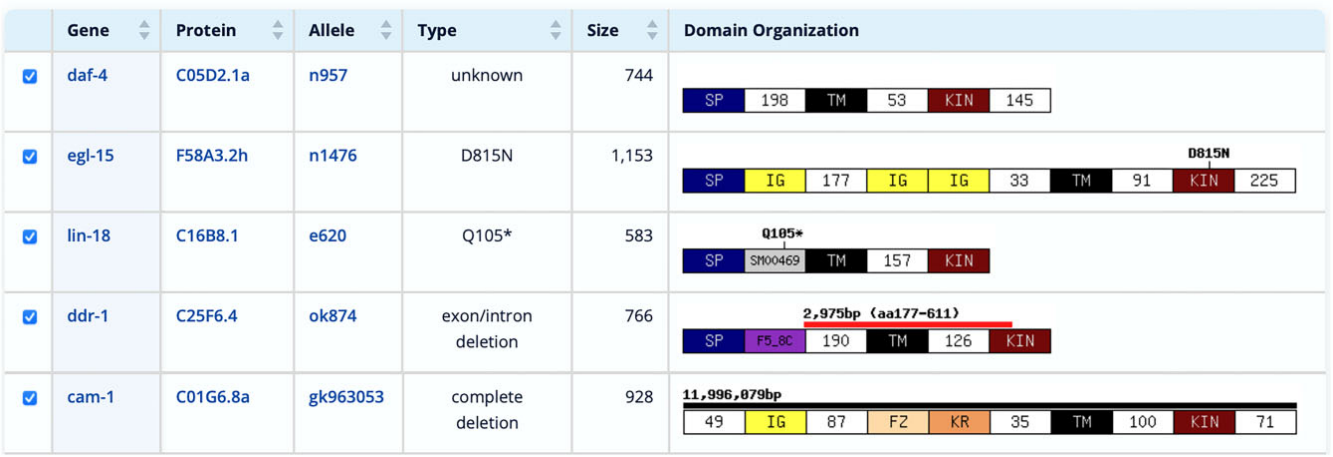
Result Table of the mutation database showing selected mutations in transmembrane kinase receptors, including an allele with no molecular data in WormBase (n957), a missense mutation (n1476), a nonsense mutation (e620), a deletion affecting part of a single gene (ok874), and a large deletion affecting multiple genes (gk963053).

The mutation database was also updated to WormBase release WS296. This release contains 1 ,839, 296 ‘variants’, which were filtered to keep only variants that affect the protein sequence. The GExplore mutation database contains 227, 218 alleles in 19 ,661 protein-coding genes (28 ,247 splice variants) with 13, 018 deletions, 183, 564 missense, and 10 ,016 nonsense alleles. Many well-characterized alleles are from older publications and do not have molecular data in WormBase—either because data were not available at the time of publication of the original study or because the data were never manually entered into WormBase. GExplore contains an additional 8,818 alleles without molecular information. These were identified by cross-checking the original set of alleles with molecular data from WormBase with alleles downloaded via WormMine. For alleles where molecular data were available, the location of the mutation was mapped onto the domain organization of the protein for a visual display of the location and nature of the mutations (Fig. 3). The database contains several large deletions affecting more than one gene. These can be excluded from the display. Mutations were mapped to all known splice variants of the gene, so that splice-variant specific alleles can be easily identified. For deletions, the location of the endpoints is indicated (‘outside’ the gene, in an ‘intron’ or in an ‘exon’). Deletion sizes are displayed as well. Display options include the possibility to show the protein sequence with the affected amino acid(s) highlighted.

#### Protein database

The protein database has been expanded and now contains data from 13 *Caenorhabditis* species (*Caenorhabditis becei, Caenorhabditis brenneri, Caenorhabditis briggsae, Caenorhabditis elegans, Caenorhabditis japonica, Caenorhabditis parvicauda, Caenorhabditis quiockensis, Caenorhabditis remanei, Caenorhabditis sulstoni, Caenorhabditis uteleia, Caenorhabditis tropicalis, Caenorhabditis waitukubuli*, and *Caenorhabditis zanzibari*) representing all branches of the *Caenorhabditis* phylogenetic tree. In addition, the database contains proteome data from seven other nematode species (*Brugia malayi, Oscheius tipulae, Onchocerca volvulus, Pristionchus pa cificus, Panagrellus redivivus, Strongyloides ratti*, and *Trichuris muris*). For comparison, the reference proteomes of the major ‘genetic model organisms’ (*S. cerevisiae, D. melanogaster, D. rerio*, and *M. musculus*) as well as the human reference proteome were included. For all nematode species, the database also contains the homologs in all other species represented in the database. The user can select proteins with certain protein domains or certain domain arrangements for a selected species. The output can be limited to proteins with homologs in one or more of the other species. This can be used to quickly identify either species-specific genes or evolutionary conserved genes of a certain type (e.g. kinases). The images showing the domain organization of proteins have been generated ahead of time, which significantly reduces the response times for a larger number of genes.

#### Expression databases

Over the last decade, many genome-wide expression profiles of different stages, tissues or cell types were established. Some of these datasets have their own dedicated websites, such as wormmap (https://www.vanderbilt.edu/wormdoc/wormmap/Welcome.html) for cell- and stage-specific transcripts from the modENCODE project [9], the CeNGEN website (https://www.cengen.org) for gene expression profiles of every neuron type [10] or the ‘single cell data explorer’ website (https://cello.shinyapps.io/celegans/) for ‘A lineage-resolved molecular atlas of *C. elegans* embryogenesis at single-cell resolution’ [11]. GExplore hosts the raw data for three genome-wide RNAseq expression datasets that do not have their own dedicated websites. The ‘expression (stages)’ database contains expression profiles of different developmental stages from embryo to adult [6]. The ‘expression (tissues)’ database contains tissues-specific expression profiles at the L2 stage [7] and the ‘expression (embryo)’ database contains expression profiles of the major tissues in the developing embryo at five different time points [8]. Together, these three datasets give a quick overview of the expression profile of every gene across stages and tissues. The search interface allows the user to search for genes with certain expression profiles such as enriched in the late embryo or maternally expressed (high expression in the gonad and in the early embryo). The search interface is designed to allow users to identify genes with expression profiles similar to their own gene(s) of interest.

**Figure 4.**
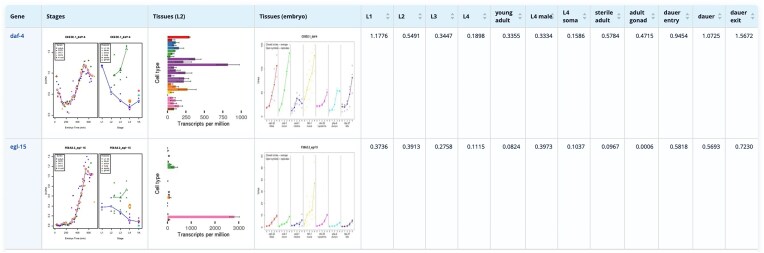
Result Table of the stage expression database showing the stage expression profiles of *daf-4* and *egl-15*, two transmembrane kinase receptors.

The display page (Fig. 4) provides a detailed view of expression profiles by combining plots and numerical values, simultaneously providing an overview and precise quantitative data. The plots allow a quick identification of genes with similar expression patterns across tissues or developmental stages, while the numerical values provide comparative information about expression levels. When integrated with mutation data, wild-type expression datasets offer a critical reference for linking genetic variation to specific tissues, pathways, or developmental stages. This combination supports bioinformatics analyses such as clustering, co-expression network mapping, and pathway enrichment, revealing functional modules and tissue-specific vulnerabilities affected by mutations. Together, these resources enable deeper insights into gene function and developmental processes. Plots and RNAseq expression values are available for download for further computational analysis.

### Web interface redesign

The original GExplore website was designed for a single database. The gradual expansion of the site and the addition of several more databases exposed the limitations of the original layout and design. We completely redesigned the website to make GExplore more user-centric and easier to use, particularly for users less familiar with the platform. The home page provides a brief description and direct links to the six databases. On the search pages, every entry field has a short help text explaining what the field accepts as input with a link to the corresponding help page subsection for a more detailed explanation. The help pages were consolidated, reorganized and rewritten with novel users in mind. They reflect the ‘workflow’ a new user would follow. After the introduction there is a description of the databases followed by an explanation of the various fields on the search pages and the various display options on the result page. A separate FAQ section helps to answer common questions and provides solutions for more complex search scenarios. We eliminated display options on the search pages. The separation of search and display options resulted in a cleaner design, which also works betters with different screen sizes.

After the search is executed, the display page provides a table with the results. The display page allows for interactive fine-tuning of the result table. Display options are now in a collapsible sidebar (Fig. 2B), which allows the full screen size to be used for the result table. The order of columns used to be fixed, but users can now drag columns around to change the order of columns allowing for a more flexible display. The column headers are shown in a fixed position, so that they remain visible when the user scrolls through the table. The ‘gene’ column is now anchored, so that it remains visible as the user scrolls horizontally through the table. The sidebar is designed to show various content, either the search parameters and the resulting list of genes or the display options or a help section. Download options are also integrated as a sidebar feature (Fig. 2B).

Taken together, the database and website updates significantly improve the usefulness of the site. GExplore is designed for experimental planning of genome-scale experiments and quick survey-type queries related to gene and protein function. The straightforward configurable output Table provides a quick overview of selected data relevant for gene function such as expression, phenotype, and protein domain organization. A distinguishing aspect is the display of mutations mapped onto the domain organization of the proteins. With these features GExplore complements data visualization tools provided by Wormbase itself (e.g. SPELL, the ‘Tissue Enrichment Analysis’ tool [12], Vennter [13], scdefg, wormcells-viz [14]) as well as others, such as the ‘*C. elegans* gene-modules analysis tools’ [15], WormEnrichr [16], or WormCat [17].

## Data Availability

The data underlying this article are available in the article and on the GExplore database website.

## References

[1] C. elegans Sequencing Consortium . Genome sequence of the nematode *C. elegans*: a platform for investigating biology. Science. 1998;282:2012–18. 10.1126/science.282.5396.2012 9851916

[2] Hutter H, Ng MP, Chen N. GExplore: a web server for integrated queries of protein domains, gene expression and mutant phenotypes. BMC Genomics [Electronic Resource]. 2009;10:529. 10.1186/1471-2164-10-529 19917126 PMC2779824

[3] Sternberg PW, Van Auken K, Wang Q et al. WormBase 2024: status and transitioning to alliance infrastructure. Genetics. 2024;227:iyae050. 10.1093/genetics/iyae050 38573366 PMC11075546

[4] Hutter H, Suh J. GExplore 1.4: an expanded web interface for queries on *Caenorhabditis elegans* protein and gene function. Worm. 2016;5:e1234659. 10.1080/21624054.2016.1234659 28090394 PMC5190144

[5] Letunic I, Khedkar S, Bork P. SMART: recent updates, new developments and status in 2020. Nucleic Acids Res. 2021;49:D458–60. 10.1093/nar/gkaa937 33104802 PMC7778883

[6] Boeck ME, Huynh C, Gevirtzman L et al. The time-resolved transcriptome of *C. elegans*. Genome Res. 2016;26:1441–50. 10.1101/gr.202663.115 27531719 PMC5052054

[7] Cao J, Packer JS, Ramani V et al. Comprehensive single-cell transcriptional profiling of a multicellular organism. Science. 2017;357:661–67. 10.1126/science.aam8940 28818938 PMC5894354

[8] Warner AD, Gevirtzman L, Hillier LW et al. The *C. elegans* embryonic transcriptome with tissue, time, and alternative splicing resolution. Genome Res. 2019;29:1036–45. 10.1101/gr.243394.118 31123079 PMC6581053

[9] Spencer WC, Zeller G, Watson JD et al. A spatial and temporal map of *C. elegans* gene expression. Genome Res. 2011;21:325–41. 10.1101/gr.114595.110 21177967 PMC3032935

[10] Hammarlund M, Hobert O, Miller DM et al. The CeNGEN Project: the complete gene expression map of an entire nervous system. Neuron. 2018;99:430–33. 10.1016/j.neuron.2018.07.042 30092212 PMC6576255

[11] Packer JS, Zhu Q, Huynh C et al. A lineage-resolved molecular atlas of *C. elegans* embryogenesis at single-cell resolution. Science. 2019;365. 10.1126/science.aax1971 PMC7428862 31488706

[12] Angeles-Albores D, N Lee RY, Chan J., et al. Tissue enrichment analysis for *C. elegans* genomics. BMC Bioinform. 2016;17:366. 10.1186/s12859-016-1229-9 PMC5020436 27618863

[13] Davis P, Zarowiecki M, Arnaboldi V et al. WormBase in 2022-data, processes, and tools for analyzing *Caenorhabditis elegans*. Genetics. 2022;220:iyac003. 10.1093/genetics/iyac003 35134929 PMC8982018

[14] da Veiga Beltrame E, Arnaboldi V, Sternberg PW. WormBase single-cell tools. Bioinform Adv. 2022;2:vbac018. 10.1093/bioadv/vbac018 35814290 PMC9258504

[15] Cary M, Podshivalova K, Kenyon C. Application of transcriptional gene modules to analysis of *Caenorhabditis elegans*’ gene expression data. G3 (Bethesda). 2020;10:3623–38. 10.1534/g3.120.401270 32759329 PMC7534440

[16] Kuleshov MV, Jones MR, Rouillard AD et al. Enrichr: a comprehensive gene set enrichment analysis web server 2016 update. Nucleic Acids Res. 2016;44:W90–97. 10.1093/nar/gkw377 27141961 PMC4987924

[17] Holdorf AD, Higgins DP, Hart AC et al. WormCat: an online tool for annotation and visualization of *Caenorhabditis elegans* genome-scale data. Genetics. 2020;214:279–94. 10.1534/genetics.119.302919 31810987 PMC7017019

